# Stepped care involving cognitive behavioral therapy for young people at clinical high-risk for psychosis in community settings: longitudinal intervention study protocol

**DOI:** 10.1186/s12888-025-07597-3

**Published:** 2025-11-28

**Authors:** Yen-Ling Chen, Sabrina Ereshefsky, Shirley Yau, Alvaro Gonzalez, Seohyun Joo, Tara Niendam, Daniel Shapiro

**Affiliations:** 1 Department of Psychiatry and Behavioral Sciences, University of California, Davis 2230 Stockton Blvd, Sacramento, CA 95817 USA; 2 Department of Psychology, National Taiwan University, No. 1, Section 4, Roosevelt Rd, Da’an District, Taipei City, 10617 Taiwan

**Keywords:** Clinical high-risk for psychosis, Stepped-care model, Community mental health

## Abstract

**Background:**

Early intervention for psychosis is associated with better clinical outcomes. The clinical high-risk for psychosis (CHRP) state is pluripotent with heterogenous outcomes. Early intervention for CHRP must focus on identifying and treating both psychosis-continuum and non-psychosis concerns. Despite the increasing focus on treatment development, accessibility of specialized interventions for CHRP is limited. Implementation of interventions for CHRP in real-life community-based settings can be challenging. Barriers include general access to affordable and affirming mental health care, limited resources and specialized workforce available in community mental health (CMH) settings, lack of community knowledge about early signs of CHRP, and difficulties engaging youth with CHRP in psychosis specialty clinics. Stepped care is a promising approach to address the above limitations.

**Methods:**

To bridge the current gap in research and practice, the SCIP-Step Program implements universal screening and a stepped-care intervention involving Cognitive Behavioral Case Management (CBCM) to identify CHRP syndromes and treat individuals presenting for care at CMH clinics. Stepped care is provided by community clinicians, starting with lower intensity treatment targeting more generalized, non-specific clinical concerns. Those who do not respond to initial stages “step up” to more intensive treatments that typically require specialized training.

**Discussion:**

The SCIP-Step Program aims to: 1) increase capacity of non-psychosis-specialty CMH agencies to identify and treat individuals with CHRP, and 2) to evaluate the clinical utility of a stepped-care intervention embedded in community settings. In this protocol paper, we outline our innovative stepped-care intervention for CHRP, approach to community partnership, and evaluation plan.

**Clinical trials registration:**

ClinicalTrials.gov, identifier: NCT06640803; registered 10/11/2024.

## Introduction

### Early intervention in psychosis improves outcomes

Early intervention (EI) is a crucial predictor of better mental health outcomes [1]. Research on early interventions for psychosis is increasingly focused on identifying early indicators of elevated risk and developing interventions that aim to decrease the rates of psychosis onset and/or mitigate its impact. Such interventions need to be flexible enough to be implemented in real-world health settings and adapt to varying clinical trajectories. Broadly implementable early intervention relies upon the following key areas: 1) screening for and detection of risk factors and indicators, such as clinical syndromes, that are sufficiently predictive of psychosis at a broad population level, 2) improving pathways into and through care so that interventions can respond to the full spectrum and heterogeneity of psychotic-continuum experiences, and 3) providing linkage to similarly targeted interventions for those who develop non-psychotic concerns [2, 3].

### CHRP is a high-risk population where EI can improve outcomes

Globally, the predominant paradigm is the clinical high-risk for psychosis (CHRP [4]) or at-risk mental state (ARMS [5, 6]) model, which identifies syndromes of attenuated, subthreshold psychosis symptoms that are predictive of a first episode of psychosis in about 25% of cases within two to three years [7]. Youths with a CHRP syndrome are more likely to develop or also meet criteria for non-psychotic mental health outcomes [8, 9]. Thus, the CHRP state is considered a pluripotent risk state, characterized by a range of symptom trajectories. Early intervention for CHRP applied prospectively then needs to focus on identifying and treating both psychosis-continuum and non-psychosis risk [10, 11] and be able to link youth with intervention programs that can treat the differing conditions they might develop.

### CHRP intervention must address a variety of mental health outcomes

Conceptually, the pluripotency and heterogeneity of the CHRP state presents challenges for real-world treatment programs. First, it has made it difficult for intervention research to identify which specific interventions are most effective in improving CHRP outcomes across the population. For example, evidence supports the effectiveness of integrated programs that offer psychosocial therapy (e.g., cognitive behavioral therapy, groups, family-based therapy), pharmacological treatment, educational and/or vocational support, and case management [12]. In some cases, the therapy uses a modularized component-based approach (e.g., engagement, assessment, safety, psychoeducation, social skills, family involvement) [13]. While these formats have the flexibility needed for the diversity of concerns with which individuals with CHRP present [13], intervention research has not yet resulted in consensus on which specific interventions are most effective in improving CHRP outcomes or contribute uniquely to this improvement [14, 15]. Second, the prevalence rate of currently defined CHRP syndromes is about 19.2% in clinical samples, including individuals accessing physical and mental health services across primary care, specialized mental health care, emergency, forensic, and school or college counseling settings [16], which means that most community-based mental health systems would not have the capacity to serve the entire CHRP population if they were able to identify them. Third, many CHRP programs in the United States are embedded within research clinics or exist as part of specialized services for those experiencing recent onset psychotic symptoms [17], each of which presents its own barriers to engaging this large population. Therefore, accessibility to CHRP care in the community population is limited. In those who do have access, the heterogeneity of clinical presentations presents additional complications. For example, some youths present with relatively low impact symptoms that they manage within their existing support networks while others may present with high impact attenuated positive or comorbid symptoms that require daily or acute support.

Workforce factors also present practical challenges for real-world implementation of CHRP care. Specialized training in evidence-based practices, such as cognitive behavioral therapy (CBT) and assessment skills to accurately identify CHRP syndromes, are costly to train to fidelity and often inaccessible in some mental health disciplines and training programs [18]. Retaining an experienced and specialty-trained workforce in community settings is an additional barrier [19]. Given pluripotency, health systems need a workforce that is able to treat varied concerns with specific evidence-based skills to respond to the potential CHRP trajectories or can cohesively link youth to other specific services. Finally, implementing any new evidence-based practice within an extant health system is a challenge. Each system has unique workflows, cultures, traditions, and practices. Making changes takes both an openness to evaluating and challenging these practices and power structures, as well as significant time, focus, and resources that may also impact other areas of the health system.

### Stepped care as potential solution

Each of these challenges shapes what can be implemented in real-life clinical settings and highlights the importance of developing EI strategies that are both conceptually and practically suited for this population *in those settings*. Stepped care has potential to be both by addressing the varied clinical trajectories of those who present for care with a CHRP syndrome *and* the complexities of implementing specialized CHRP care in real-life community-based settings. Addressing the varied needs and outcomes in the CHRP state, a stepped-care approach is particularly suited because it is based on a clinical staging framework [20]. Clinical staging, adopted from other areas of medicine, uses a dimensional approach to conceptualize psychopathology and focuses on both transdiagnostic and specific risk factors for mental disorders [21, 22]. Early on in the course of clinical presentations, symptoms are less specific and may be more heterogenous in terms of needs – similarly, early stages in the clinical staging model focus on milder, less specific symptoms (i.e., transdiagnostic approach) and a less intensive treatment approach [20, 21] that can be provided by a less specialized, less costly, and more available workforce. This early stage of treatment can expeditiously triage limited resources by starting to treat non-specific clinical and non-clinical concerns (e.g., anxiety, mood, school difficulties) and can be implemented by a broad array of providers. For some, needs will increase as syndromal and diagnostic specificity increase over time [23], while those most likely to remit or benefit from generalized mental healthcare will see their support needs lessen over time. Therefore, at more advanced stages of treatment, the focus becomes more specific and requires more specialized intervention to target recurrent, persistent symptoms associated with increased distress and/or impact to important areas of life [20, 21]. This adaptive and sequential approach triages more intensive and specialized interventions for those who do not respond to earlier stages of treatment. From a health system perspective, stepped care is a proactive approach, with an emphasis on EI and expert monitoring as the first step to treatment. Preliminarily data on a stepped-care model implemented at the health systems level have demonstrated the acceptability [24] and benefits [25] of the CHRP stepped-care approach.

### Stepped-care involving Cognitive Behavioral Case Management (CBCM) as a model for CHRP care in community-based settings

Cognitive Behavioral Case Management (CBCM) was initially developed for the NEURAPRO trial [26, 27]. It was based on the standard manualized clinical program used in Orygen’s Personal Assessment and Crisis Evaluation (PACE) clinic in Melbourne, which was used to inform clinical high risk services nationwide [28] but shaped to be implemented by front-line level providers in generalist adolescent mental health settings. Previous studies outside the US have supported the feasibility of implementing CBCM in generalized youth community health settings with generalist providers and demonstrated robust clinical improvements compared to baseline [27, 29]. The current Sacramento Clinical high-risk Intervention for Psychosis Stepped care Program (SCIP-Step Program) has adapted CBCM and stepped-care for US-based settings based on results of two previous investigations. First, stepped care using CBCM was evaluated in a sequential multiple assignment randomized trial of fluoxetine and CBCM conducted at PACE and in primary youth mental health care clinics in Australia [24, 30, 31]. The authors found that a sequential approach to treatment, starting with the least specialized interventions and moving to more targeted and intensive interventions (CBCM and fluoxetine) in those whose symptoms did not remit, was acceptable to service users and associated with mild to moderate improvements in functioning and symptoms [24, 30].

The second investigation was conducted at the UC Davis Early Psychosis Program (Early Diagnosis And Preventive Treatment Clinic; EDAPT) in collaboration with Orygen in Melbourne. EDAPT served as a Staged Treatment in Early Psychosis (STEP) study feasibility site. This study aimed to replicate the broad structure of STEP in an American healthcare setting. Results indicated that that it could be implemented in the US [32]. In addition, once youth at CHRP were engaged in the clinic, they accepted stepped-care using CBCM when alternatives were available, tolerated the intervention, and the intervention was associated with positive outcomes [32]. However, some key aspects of the US healthcare setting that precluded access to the intervention on a large scale were also identified. Specifically, waiting to initiate assessment for CHRP and to offer stepped care until after youths were referred to and reached our specialty early psychosis service was a major barrier. The complicated and sometimes lengthy process of referral out of community-based clinics at which youths initially sought care led to the loss of contact with a large proportion of those who could potentially benefit. Limitations in the specialty early psychosis clinic’s ability to provide higher levels of care, lower levels of care, or evidence-based services for comorbid concerns also precluded many potentially at-risk youth from enrolling in our clinic and thus from being offered stepped-care. Finally, more specific to the STEP trial, medication preferences also prevented a number of youth from joining a study that involved randomizing medication.

### The current study will tailor stepped care using CBCM to a county-wide community health system in the US

The results of the US STEP implementation were used to shape the SCIP-Step Program to better meet the needs and structure of participating community mental health (CMH) agencies. Specifically, SCIP-Step implements screening and identification of CHRP syndromes in community-based clinics where youth typically first seek care, which may be more accessible, affordable, and affirming than a specialty psychosis setting. This also reduces hurdles that result from needing to navigate additional referrals or handoffs. Implementing the early steps of care in these generalized settings also supports more flexible treatment for youths who need a broader range of services or service intensity or those who no longer meet CHRP criteria during care. All clinics chosen for SCIP-Step provides a flexible integrated treatment (FIT) approach, which is moderate intensity, team-based, and supports care that can be provided at-home or in the community. One of the participating clinics specializes in providing evaluation and treatment of child maltreatment. Because more than 50% of youth in the original trial using CBCM sought additional treatment after the active phase of therapy [29], we extended the duration of care to two years. We also include consultation with community medication providers because preferences and concerns about medication were present in initial US STEP implementation trial. Finally, by focusing our limited psychosis specialty care resources on conducting assessments, training and supporting community clinicians in providing treatment, these resources can be used more efficiently. More specialized interventions and intensive care are provided only in more advanced steps of stepped care. This approach allows for a much broader population of youth to be screened, identified, and treated than if specialty clinics were responsible for delivering and implementing all services themselves. Thus, the stepped care model both preserves limited CHRP specialized skill and knowledge and seeks to build it in the general CMH workforce.

The current study aims to evaluate the implementation and clinical utility of SCIP-Step Program with youth identified as CHRP in CMH agencies serving the same community (Sacramento County, CA). This study partners with CMH agencies to maximize the ecological validity of the stepped-care model in real-world settings. In this protocol paper, we outline the design of the SCIP-Step Program, a community-embedded, stepped-care intervention program for CHRP. The goal of the SCIP-Step Program is two-fold: 1) to increase the capacity of non-psychosis-specialty CMH agencies to identify and treat youth experiencing CHRP symptoms; and 2) to evaluate the ability to successfully implement a stepped-care intervention that triages specialized interventions for CHRP symptoms in CMH agencies.

## Method

### Study population

#### Youth participants

This project is conducted in partnership with the Sacramento County Department of Health Services – Behavioral Health Services (SCBHS), which supports, coordinates, and funds healthcare for individuals who have Medicaid (Medi-Cal) insurance or are uninsured. All youth in this project are help-seekers who present for care at one of the six SCBHS-contracted CMH agencies at which the study is being conducted. Additional inclusion criteria include: (a) Sacramento County residency and eligibility for SCBHS services, (b) age 12–25, (c) criteria met for CHRP, and (d) ability to understand and consent/assent to the study procedures. If youth are eligible and agree to enroll in the SCIP-Step Program, bilingual study staff can complete consent/assent in English, Spanish, and Chinese with consent/assent forms available in these three languages. For minors, informed consent is obtained from their legal guardian, and assent is obtained from the youth. UC Davis medical interpreting services provides interpreters to complete the consent/assent process when guardian prefers a different language. Exclusion criteria include: (a) IQ < 70 as determined by participating CMH clinic or (b) urgent clinical needs that lead to transfer out of the CMH clinic at which care is being sought.

#### Treatment sites

Sacramento County recommended potential CMH agencies, who then met with SCIP-Step leadership to learn about the program and mutually decide if SCIP-Step would benefit their community and families. The CMH sites provide services that target a range of severity (low, moderate, specialized trauma care) and with treatment models in which youth can receive care for two years or more. The SCIP-Step Program sites include Capital Star Community Services, HeartLand Child and Family Services, River Oak Center for Children, Turning Point Community Programs, and UC Davis Child and Adolescent Abuse Resource and Evaluation Center (CAARE).

#### Program providers

All providers involved in clinic intakes at participating CMH agencies receive training on CHRP symptoms and screening procedures. To identify potential therapy providers, CMH agency leadership select therapists and case managers who either have interest in working with the CHRP population or are selected based on needs of their clinical teams. These potential therapy providers are invited to attend a two-day workshop focusing on CBCM and the stepped-care intervention. After completing the two-day workshop, providers choose to formally enroll in the SCIP-Step Program by signing a consent form. All service users who meet CHRP criteria and consent to SCIP-Step are then assigned to these consented clinicians either at project consent or before the service users reach the CBCM step at six-months. Providers in these agencies are primarily Clinical Social Workers (CSWs) and Marriage and Family Therapists (MFTs) but there are also pre-doctoral psychology trainees. Providers in these agencies have predominantly been in the mental health field for less than 10 years and not yet independently licensed (e.g., Associate MFT and CSW, psychology interns [33]).

### Measures and materials

#### Prodromal Questionnaire-Brief (PQ-B)

The PQ-B [34] is a screener developed to identify those who might be experiencing psychosis spectrum symptoms and thus, would benefit from further assessment. It has been validated in adolescents and young adults, as well as across gender, race/ethnicity, and various languages [35–39]. It consists of 21 self-report items assessing past month positive psychosis-continuum symptoms experienced. For each endorsed item, individuals also rate their distress level from 1 (*strongly disagree*) to 5 (*strongly agree*). The PQ-B was developed as a screening tool – not a diagnostic tool – and thus, has high sensitivity but low specificity [34]. A total distress score between 18 - 24 has been suggested as a validated cut-off in CMH settings [39, 40]. In this study, we use a total distress score ≥ 20 to identify positive screens who should move on to additional assessment.

#### Abbreviated clinical structured interview for DSM-5 attenuated psychosis syndrome (mini-SIPS)

The Mini-SIPS [41] is an abbreviated version of the Structured Interview for Psychosis-risk Syndromes (SIPS;[42]), a gold-standard, semi-structured interview for identifying CHRP via attenuated psychosis syndrome (APS). The Mini-SIPS is administered by trained clinicians who achieved inter-rater reliability on the measure. It was developed in order to better meet the needs of clinical settings: the format was distilled from the original SIPS to focus exclusively on positive symptoms (hallucination-like, delusion-like, disorganized communication) and identifies where on the continuum an individual’s experiences fall – normal range, attenuated range, or psychotic range – rather than the prior Likert scoring system.

#### Substance Abuse and Mental Health Services Administration (SAMHSA) National Outcome Measures (NOMS)

The NOMS [43] is a survey tool that collects information on demographics and past month education/employment, criminal justice involvement, social connectedness, housing, mental health symptoms, services received, and perception of services. The NOMS tool is required of all SAMHSA-funded projects to track progress.

#### Global functioning: social and role scales (GF:S/R)

The GF:S/R [44] is comprised of separate social [45] and role [46] domain ratings, distilled and modeled after the Global Assessment of Functioning [47, 48]. It includes a semi-structured interview, which was validated for use in CHRP and early psychosis populations [49, 50]. The social scale assesses an individual’s quantity and quality of social relationships (e.g., friends, family, age-appropriate intimate relationships), whereas the role scale assesses an individual’s performance and level of support needed in their respective role (school, work, or homemaker). For both social and role scales, scores range from 1 (*extreme dysfunction*) to 10 (*superior functioning*). Only the past month rating is collected by the SCIP-Step team as part of this project at each assessment point.

#### Modified Colorado Symptom Index (MCSI)

The MCSI [51] is a 14-item self-report questionnaire measuring the past-month frequency and severity of a broad range of psychological symptoms, including depression, anxiety, cognitive symptoms, and positive symptoms of psychosis. Participants rate the frequency of experiences from 0 (*not at all*) to 4 (*at least every day*). It has been used to assess treatment response by tracking reduction in psychiatric symptoms over time [51] and is currently included in the Early Psychosis Intervention Network (EPINET) Core Assessment Battery [52].

#### Child and adolescent trauma screen (CATS)

The CATS [53] is a self-report screening tool for 7–17 year old youth, measuring potential traumatic events (15 items), posttraumatic stress symptoms (20 items), and psychosocial functioning (5 items) based on the DSM-5 criteria for posttraumatic stress disorder (PTSD). Participants first rate whether they have experienced any traumatic events using a binary yes/no scale, and then they rate the frequency of posttraumatic stress symptoms in the past two weeks related to the event that is most distressing to them, from 0 (*never*) to 3 (*almost always*). Finally, participants provide ratings on whether symptoms have interfered with their functioning (e.g., school or work, family relationships) using a binary yes/no scale.

#### Life events checklist for DSM-5 and PTSD checklist for DSM-5 (LEC-5 and PCL-5)

The LEC-5 [54] is a 17-item self-report screening tool for individuals aged 18 or above to assess lifetime exposure to potential traumatic events. Participants rate whether and how they have experienced any traumatic events in their entire life, noting *happened to me*, *witnessed it*, *learned about it*, *part of my job*, *not sure*, or *doesn’t apply*. The PCL-5 [55] is a 20-item self-report measure for posttraumatic symptoms based on the DSM-5 criteria for their most distressing endorsed trauma. Participants rate how much they have been bothered by PTSD symptoms in the past month from 0 (*not at all*) to 4 (*extremely*).

#### Systemic Clinical Outcome and Routine Evaluation index of family functioning and change (SCORE-15)

The SCORE-15 [56] is a 15-item self-report measure assessing family well-being, including family strengths, family difficulties, and family communication. Family is defined by the individual and includes “chosen” family. Participants rate items by answering how well each description describes their family, from 1 (*describes us: very well*) to 5 (*describes us: not at all*). The SCORE-15 has been used in family therapy to assess different aspects of family process and functioning.

#### SCIP-Step treatment progress self-assessment

In partnership with the CBCM treatment developers, the SCIP-Step Program developed a self-assessment of treatment progress and satisfaction that is similar to what is administered in the Headspace clinics. This assessment tool consists of (a) a one-item Global Impression Scale [57, 58], which has been modified for self-report and to reword anchors to refer to “symptoms” instead of “illness,” and (b) a series of Likert scaled items: 0 (*completely disagree*) to 100 (*completely agree*) about progress in various areas of well-being and important areas of life, satisfaction with different services received, and perceptions of usefulness of care and services received. Participants also indicate which services they have engaged with during the past six months.

### Design and procedure of the stepped-care model

#### Broad structure

**Fig. 1:**
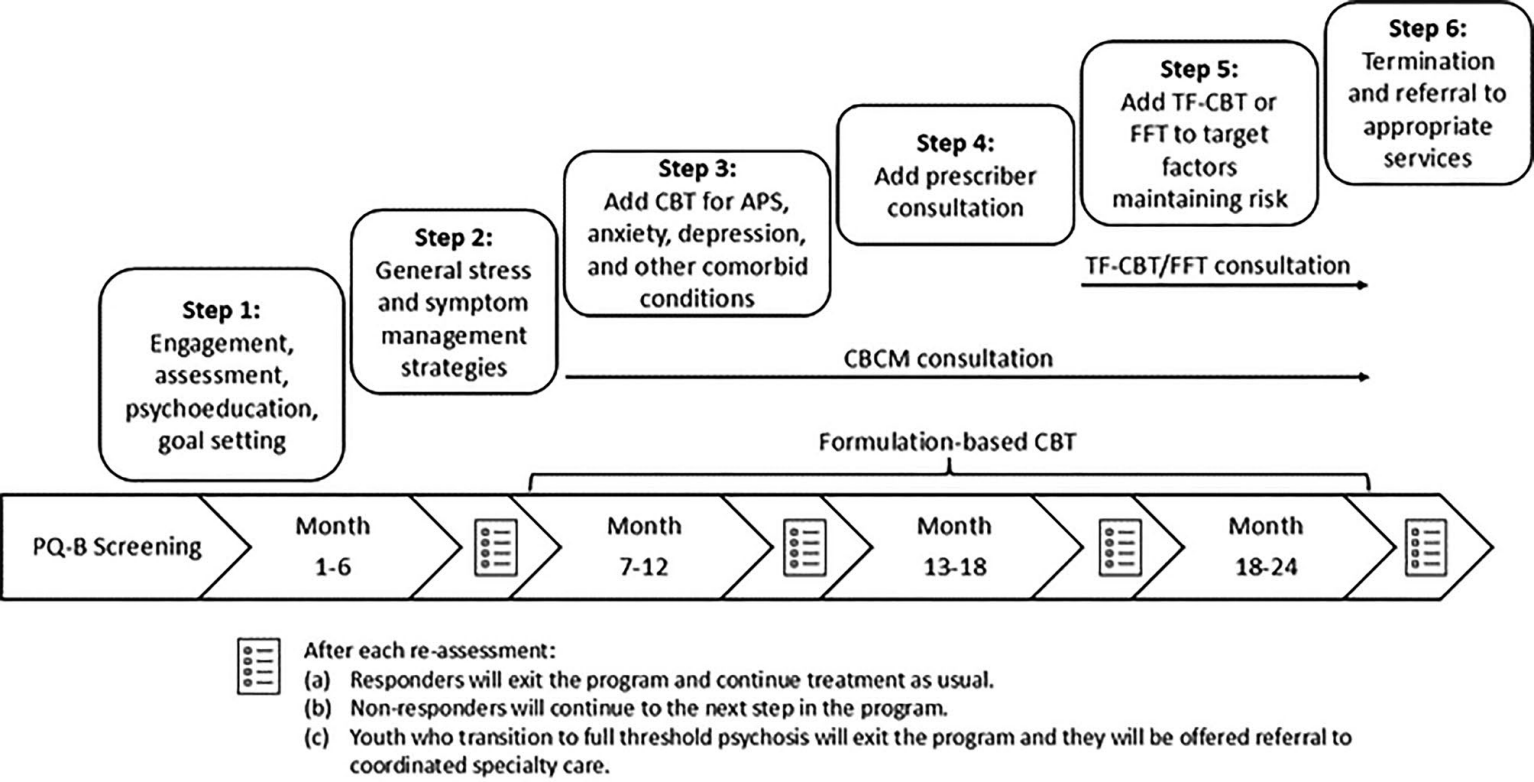
SCIP-Step program’s stepped-care model. *Note.* APS: attenuated psychosis syndrome; CBCM: cognitive behavioral case management; CBT: cognitive behavioral therapy; FFT: family-focused therapy SCIP-Step: Sacramento clinical high-risk intervention for psychosis stepped Care; TF-CBT: trauma-focused cognitive behavioral therapy

The SCIP-Step Program utilizes a sequential approach to treatment (Fig. 1). Our model starts with universal screening as secondary prevention at CMH centers with the aim of screening for psychosis-risk at the help-seeking population level in Sacramento County. Conducting universal screening at CMH centers can better reach the population in need because those with unidentified psychosis-risk syndromes tend to present to generalized settings first [59]. Additionally, universal screening results in the identification of more youth with psychosis-spectrum symptoms than indicated screening based on clinician judgement [60]. Broadly, those who meet CHRP criteria and agree to enroll in stepped care complete SCIP-Step assessments every six months to determine whether they “step up” to more intensive and specialized care, “step down” to general community care, or “step over” to more specialized care for other developing concerns. Specialized care can include direct transition to EDAPT if youths develop psychosis or do not improve within two years of stepped care – EDAPT is the only coordinated specialty care (CSC) clinic for early psychosis in Sacramento County. To triage the limited early psychosis resources in this region, more intensive and specialized interventions are reserved for those with more severe symptoms or those who do not improve with less specialized interventions. Within the SCIP-Step Program, screening and intervention is delivered by CMH providers, while the SCIP-Step team leads specialized CHRP assessments and psychoeducation feedback sessions with identified youth and their support persons, in addition to providing intensive training and at least monthly provider consultation.

#### Cognitive behavioral case management (CBCM)

In its original form, CBCM (The [28]) was comprised of six modules: stress management, depression/negative symptoms, positive symptoms, basic symptoms, and other comorbidities (e.g., anxiety). With ongoing consultation from CBCM developers, new materials were developed for US-based CBCM implementation keeping a similar structure, with a few notable differences – we added a psychoeducation module, removed the basic symptom module, and expanded the anxiety module. CBCM is intended to be flexible. In the initial steps, there are more general interventions that can be delivered by almost any team member and transitions to a formulation-driven approach at later steps that requires more provider specialization and individualization.

#### Assessment procedures

Assessments are conducted at baseline, 6-, 12-, 18-, and 24-months. Table 1 displays the assessment schedule of the SCIP-Step Program, including measure names and timepoints of assessment. Baseline assessments consist of the following: PQ-B, Mini-SIPS, NOMS, MCSI, GF:S/R and SCIP-Step Treatment Progress Self-Assessment. The PQ-B is used for screening psychosis risk and completed for all individuals going through the screening stage. The Mini-SIPS is administered to those who screen positive on the PQ-B to determine diagnosis of Attenuated Psychosis Syndrome and eligibility for SCIP-Step Program. See Screening/Identification for more details on the PQ-B and Mini-SIPS. The NOMS, MCSI, GF:S/R, and SCIP-Step Treatment Progress Self-Assessment are only administered to individuals who are eligible for the SCIP-Step Program after enrollment.

**Table 1:** SCIP-Step assessment schedule

Domains	Measures	Method	Baseline	6-Month	12-Month	18-Month	24-Month	Discharge
Symptoms	Prodromal Questionnaire-Brief (PQ-B; [34])	Self-report	X (screening)					
	Abbreviated Clinical Structured Interview for DSM-5 Attenuated Psychosis Syndrome (mini-SIPS; [41])	Interview	X	X	X	X	X	
	Substance Abuse and Mental Health Services Administration (SAMHSA) National Outcome Measures (NOMS; [43])	Interview	X (enrollment)	X				X
	Modified Colorado Symptom Inventory (MCSI; [51])	Self-report	X (enrollment)	X	X	X	X	
	Child and Adolescent Trauma Screen (CATS [53]; or PTSD Check List-Version 5 (PCL-5; Weathers et al., 2013)^a^	Self-report				X	X	
	The Systemic Clinical Outcome and Routine Evaluation Index of Family Functioning and Change (SCORE-15 [56]; ^a^	Self-report				X	X	
Impact on important areas of life	Global Functioning Social and Role Scales (GF:S/R [44–46];	Interview	X (enrollment)	X	X	X	X	
Self-perceived progress and satisfaction with care	SCIP-Step Treatment Progress Self-Assessment^b^	Self-report	X	X	X	X	X	

Note.^a^Enhanced assessment; ^b^Measure developed by the SCIP-Step program for this project

At each re-assessment timepoint (i.e., 6-, 12-, 18-, and 24-month re-assessments), the Mini-SIPS is used to assess treatment response and progression to subsequent steps. The MCSI, GF:S/R, and SCIP-Step Treatment Progress Self-Assessment are also administered to all individuals at each re-assessment timepoint for outcomes tracking. The NOMS, required for all SAMHSA-funded projects, is completed at six-month re-assessment and discharge (whenever an individual exits the project). At the 18-month re-assessment, enhanced evaluation is implemented via CATS or LEC-5 and PCL-5, as well as, SCORE-15 to identify additional clinical concerns that should be integrated into later steps of the stepped-care model. At the 24-month re-assessment, CATS or LEC-5 and PCL-5, as well as, SCORE-15 are administered again as a post-assessment to evaluate progress after evidence-based treatment for trauma symptoms and family therapy.

#### Screening/Identification

The SCIP-Step team partnered with leadership in each community clinic to co-develop screening workflows that integrate with extant intake procedures. These procedures were integrated into a screening training provided to clinic staff: topics include an overview on CHRP and SCIP-Step, the PQ-B, and referral procedures. In general, universal screening is completed as part of the clinic’s intake procedure with new service users. For existing service users who are already enrolled in the community clinic prior to the implementation of the SCIP-Step Program, their clinicians may also complete the PQ-B screening when there is concern about psychosis risk (i.e., indicated screening). For most agencies, the screening is completed on an electronic device (e.g., project-funded tablets, personal device) linked to Research Electronic Data Capture (REDCap), a secure data collection platform and repository. No protected health information is stored in REDCap. Paper and pencil screening can also be completed and subsequently entered into REDCap. Individuals are informed that screening involves sharing de-identified information outside of their clinic. Individuals can decline screening if they are not comfortable with proceeding. Once a youth agrees, initial screening pages gather de-identified information and eligibility parameters, partially to help the SCIP-Step team match screening results with referrals, as well as gather more broad characteristics on all screening participants. English and Spanish [36] versions of the PQ-B are provided during the training and embedded within REDCap. The PQ-B is available in other validated languages upon request. REDCap automatically scores the PQ-B and sends results to the SCIP-Step team. Built-in scripts are provided to CMH clinicians for guidance on what to do after screening. All screened individuals are assigned a study ID. Youth who screen positive (PQ-B total distress score ≥ 20) are offered an opportunity to complete a CHRP specialized assessment. Those who give permission to complete this assessment are formally referred to SCIP-Step. For those who screen positive but decline further assessment, clinicians complete a de-identified referral document specifying the service user declined further contact.

**Fig. 2:**
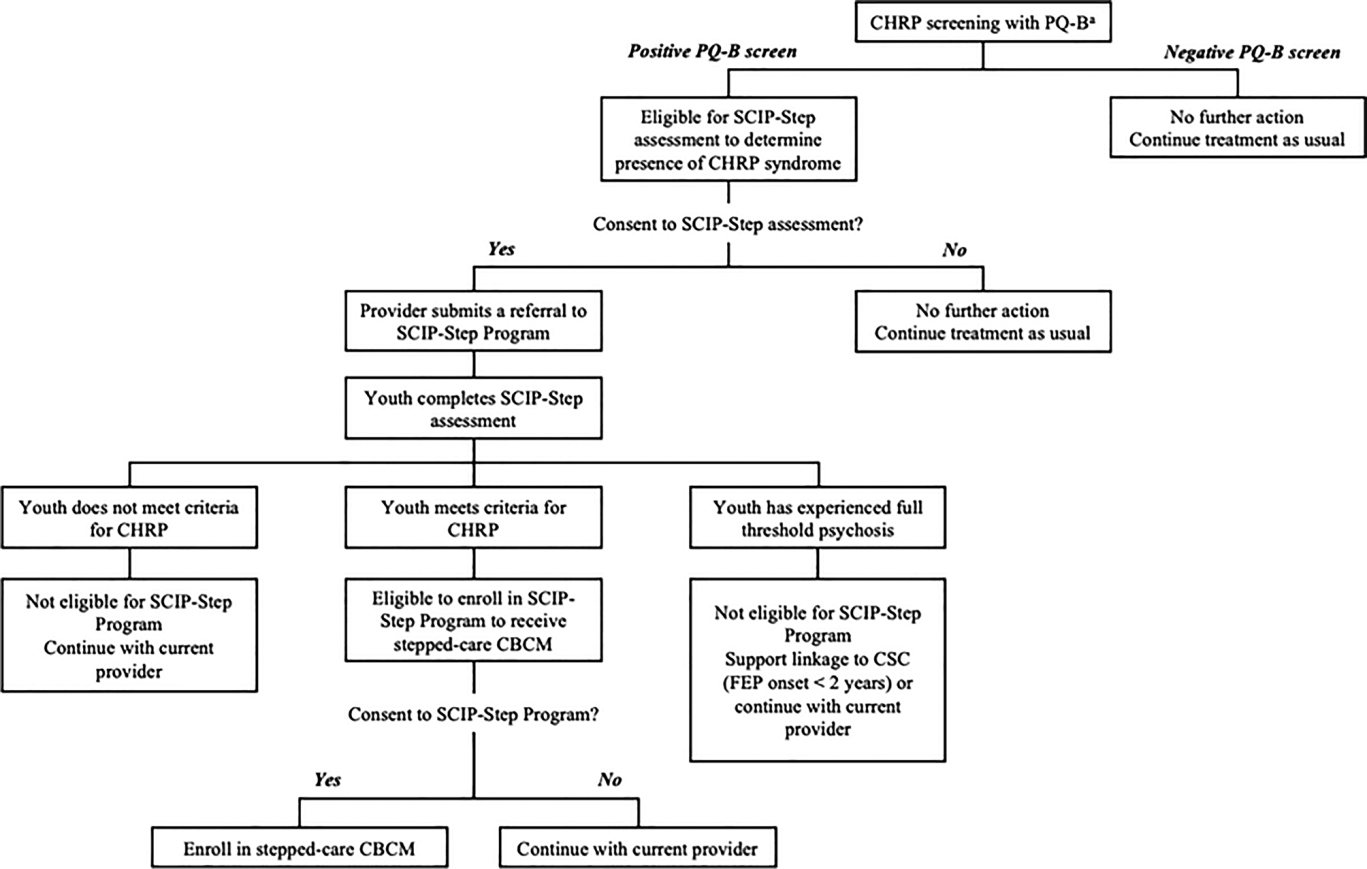
Flow diagram of screening and assessment before entry into the SCIP-Step program. *Note.* CHRP: clinical high-risk for psychosis; CMH: community mental health; FEP: first episode psychosis; PQ-B: Prodromal Questionnaire – Brief; SCIP-Step: Sacramento Clinical high-risk Intervention for Psychosis Stepped Care. ^a^ For new service users, universal screening is completed at intake. For existing service users, indicated screening is completed when there is concern about psychosis risk

Once the referral is received, the SCIP-Step team schedules and completes a baseline evaluation using the Mini-SIPS. Assessments are completed at a location most convenient to the service user and their family, including at their community clinic, another preferred community location (e.g., home, school), or via telehealth. Decisions regarding eligibility and recommendations are discussed within internal SCIP-Step assessment group supervision. Subsequently, feedback is completed with all participating individuals, providers, and support persons, as applicable. During the initial feedback session, those who meet criteria for CHRP are invited to enroll in SCIP-Step. If an individual agrees to enrollment, SCIP-Step staff review study consent/assent and complete baseline measures (see Measures section). Youth then receive compensation and start receiving stepped care as soon as they are linked with a consented provider. If youth do not have psychosis-continuum experiences, brief feedback is completed and youth are recommended to remain with their CMH clinician without modifying treatment. For youth who are identified as having threshold psychosis, they may be referred to EDAPT for specialty early psychosis care if psychosis has been present for less than two years; if psychosis is present for more than two years, the feedback session can be used to determine the most appropriate and beneficial course of intervention. Figure 2 displays a diagram showing how individuals are triaged at the screening and assessment phases based on their level of need and clinical resources.

#### Stepped care (Step 1 to Step 6)

Figure 1 displays the stepped-care design used in the SCIP-Step Program. At the time of enrollment, individuals begin with the least specialized intervention. Youth who enter via indicated screening later in their established care may begin at a later step. The first two steps, which approximate treatment as usual, occur over the first six-months of care after intake; these steps aim to engage youth in their CMH clinic, begin generalized interventions that are most likely to benefit most, and can be provided by anybody on the team (e.g., case managers or therapists). Step 1 focuses on assessment feedback and psychoeducation, getting settled into the clinic, needs assessment to begin case management and address social determinants of health, treatment goal identification, and general engagement with the team. During Step 1, focus is also on initiation of aspects of team-based care at community clinics (e.g., school support) and “enhanced monitoring,” comprised of identifying a CHRP syndrome and associated psychoeducation (using the CBCM psychoeducation module). Once broad goals are set for care, individuals begin with standard treatment as they would typically in their community clinic. Step 2 is comprised of theoretically-linked skills from the CBCM stress-management module, such as developing a personalized subjective units of distress thermometer, coping skills and plans, and problem-solving strategies. Movement from Step 1 to Step 2 is intended to be flexible and to occur when the youth and clinician are ready. These interventions require minimal CHRP-specialized training, can apply to any mental health concerns, and do not rely on any CBT formulation or specialization. The rationale is that many youths with CHRP remit within the first year after identification [61], and they may be the most likely to benefit from generalized mental health interventions. Furthermore, it often takes time to build relationships with treatment providers, and in many cases, addressing resource needs are a higher priority in stabilizing clinical concerns than beginning therapy. If individuals still meet CHRP criteria after six months, they “step up” to a more targeted and intensive CHRP intervention (Step 3), which includes initiation of formulation-based CBT modules and consultation groups for therapy providers. This typically coincides with use of the CBCM symptom-based modules (depression/negative symptoms, anxiety, attenuated psychosis syndrome) as indicated. At 12-months, youth who still meet CHRP criteria “step up” (Step 4) with initiation of psychiatric consultation groups comprised of clinic prescribers working with an EDAPT prescriber consultant. In Step 4, therapy providers continue formulation-based CBT intervention and consultation groups.

By 18-months (Step 5), the individuals who still meet CHRP criteria are likely more homogenous, thus, there is more confidence and concern about psychosis-risk. Treatment at this timepoint aims to assess and treat what additional factors may be maintaining risk for psychosis that were not addressed in previous steps. At 18-months, the reassessment includes additional measures to assess family conflict and trauma, strong predictors in those who develop psychosis [62, 63]. If either is identified, additional evidence-based practices (EBP) for these concerns – family-focused therapy (FFT) for individuals at clinical high risk [64] or trauma-focused cognitive behavioral therapy (TF-CBT [65]) – are added as part of the stepped-care intervention. Clinicians then begin attending consultation groups for either FFT or TF-CBT. If these factors are not present, service users could opt to initiate CBT for CHRP (CBTp) at EDAPT or continue with their current CMH provider. At 24-months, individuals who do not respond to all previous treatment components and continue to present with ongoing CHRP symptoms will be referred to services that best fit their current clinical picture, which can include coordinated specialty care (CSC) at EDAPT (Step 6). Clinicians are asked to fill out a CBCM checklist after each session indicating which step they believe therapy is in and what types of interventions they used during the session. If clinicians and service users feel that they need to move into a more advanced therapy step, for example if they are ready to work on depression before 6 months occur or an EBP like TF-CBT is indicated before 18 months, they have this flexibility and will indicate this on their CBCM checklist.

Reassessments occur every six months and determine movement between steps. For the purpose of transition from one step to another, response to treatment is operationalized as “no longer meeting current CHRP criteria” according to the mini-SIPS. Non-response is the presence of CHRP symptoms in the past month, and these individuals “step up” to more intensive intervention. At any step, if an individual no longer meets CHRP criteria, they “step down” and exit the program, which entails staying with their current provider for treatment as usual. If an individual transitions to full-threshold psychosis at any point in the stepped-care intervention, they are offered referral to EDAPT for CSC to reduce their duration of untreated psychosis (DUP), defined as the period between the onset of full-threshold psychosis and the initiation of first adequate treatment [66]. DUP is associated with poorer prognosis [67]. Therefore, the current design of the stepped-care intervention also aligns with the vision of early intervention to reduce DUP, as well as, improve relevant clinical outcomes [68].

#### Ongoing support and partnership

The SCIP-Step Program offers ongoing implementation support throughout the intervention. Each treatment site receives ongoing training on PQ-B screening for psychosis risk and referral to the SCIP-Step Program for further eligibility assessment. Ad-hoc consultation during the screening and assessment process are available upon request from community providers. Providers have access to the training resources and materials through a cloud-based shared drive, including treatment handouts created by the SCIP-Step team. Therapists working actively on CBCM with enrolled participants are required to attend group consultation, at least monthly, hosted by the SCIP-Step team. Prescribers working with youth at the 12-month timepoint are asked to attend monthly consultation group. To enhance partnership between the SCIP-Step team and partnered sites, SCIP-Step leadership and clinic leadership meet biweekly to assess and modify workflows; support reconciliation of screening, intakes, and referrals – working toward universal screening; ensuring all enrolled participants are linked with providers who are trained in the intervention and enrolled in SCIP-Step; and planning and supporting training and consultation. Additional opportunities for knowledge transfer and partnership include SCIP-Step team members being present in site clinical meetings, holding “Question and Answer” (Q&A) sessions, and sharing aggregate outcomes data with providers and clinic leadership.

## Measuring outcomes of SCIP-Step implementation

Outcomes in this study will include various indicators of program implementation and applicability organized in the RE-AIM framework [69, 70]. First, we will report the universal screening rates and number of clinicians trained in screening to assess the *reach* of the program in the community. We will also examine the characteristics of youth involved in the program to assess the representativeness of the study sample to the targeted populations. *Effectiveness* of the program will be measured by rates of engagement and withdrawal of both service users and clinicians, clinical outcomes, including remission and transition rates, as well as, self-report symptom severity and areas of improvement using evidence-based measures (summarized in *Measures and Materials)*. We will also assess participants’ self-report experiences of receiving care as part of the SCIP-Step Program and change in these scores at re-assessment timepoints. Regarding *implementation*, we will collect session checklists from community providers to measure fidelity to the various components of CBCM in the stepped-care model. Throughout the course of the study, several quality improvement (QI) initiatives will be carried out, including quantitative surveys and qualitative interviews with partnered clinicians. These QI initiatives aim to collect feedback from community partners regarding their perceptions and actual experiences delivering stepped care in CMH settings. Broadly, we employ two types of QI initiatives. One is to improve the training provided by the SCIP-Step Program; the other one is to explore potential barriers and facilitators through a dissemination and implementation lens. Results will help to identify key mechanisms impacting implementation to help us iteratively enhance the *adoption* and *maintenance* of the stepped-care intervention by community providers. We also intend to use results to inform sustainment efforts.

### Proposed enrollment and sample size

Over the four-year study period, we plan to implement services in two CMH agencies per year over three years, for a total of six agencies enrolled at the end of year three. Estimated sample size (Table 2) was based on a previous study of screening rates using the PQ-B in Sacramento County [60] and agency census estimates provided by Sacramento County. Based on these rates, we anticipate approximately 150 youth ages 12–25 will be screened annually per site, with 2,700 youth screened by the time of study completion in September 2026. Among all youth screened, we anticipate approximately 39% (*n* = 1,053) will screen positive on the PQ-B [60]. Of those who screen positive, we anticipate approximately 68% (*n* = 717) will consent and be referred for further CHRP assessment [60]. Subsequently, from those who complete the assessment, we estimate that approximately 28% (*n* = 198) will meet criteria for CHRP, 31% (*n* = 222) for first episode psychosis (FEP) and psychosis duration longer than two years, and 41% (*n* = 297) for no psychosis [60]. Of those with CHRP, in conjunction with interest in the intervention, we anticipate enrolling 198 youth at CHRP in the SCIP-Step Program by completion of the study. Recruitment of service users is ongoing and is expected to be completed on September 29^th^, 2026.

**Table 2:** Estimated number of individuals in the SCIP-Step program

	Year 1 (2 CMH sites)	Year 2 (4 CMH sites)	Year 3 (6 CMH sites)	Year 4 (6 CMH sites)	Total
Eligible and complete PQ-B screening	300	600	900	900	2,700
PQ-B screen positive and complete CHRP evaluation	80	159	239	239	717
Experience CHRP symptoms and enroll in SCIP-Step Program for stepped care	22	44	66	66	198

Note. CMH: community mental health; PQ-B: Prodromal Questionnaire – Brief; CHRP: clinical high-risk for psychosis; SCIP-Step: Sacramento Clinical high-risk Intervention for Psychosis Stepped Care

### Proposed analyses

Prior to data analyses, all data will be screened and examined for missing values and outliers. Missing data will be addressed with appropriate statistical approaches based on their reason for missingness. Descriptive statistics will describe the characteristics of samples, using means and standard deviations (SDs) for continuous variables (or median and inter-quartile range [IQR] for non-normal distributions), and frequencies and proportions for categorical variables. Cross-sectional comparison among sub-groups will be tested with between-subject T-test or Analysis of Variance (ANOVA) for continuous outcome variables, and Chi-square test for categorical outcome variables. Mixed-effect models will be used to examine how outcomes change over time, with fixed effects for the program timepoints, nesting, and potential covariates, and random effects for subject-specific response over time. Barriers and facilitators interviews will be transcribed and de-identified and analyzed to identify common emergent themes. For purposes of QI, these will be discussed with community partners so that mechanisms underlying barriers can be identified and interventions can be selected to address them.

### Ethics and protocol registration

The Institutional Review Boards (IRB) of University of California, Davis (UC Davis; IRB # 1,989,082-7) and Sacramento County Department of Health Services approved the study. During the implementation process, any modifications that materially affect the SCIP-Step protocol are reviewed and approved by the UC Davis IRB. The SCIP-Step protocol is also registered on ClinicalTrials.gov (Identifier: NCT06640803).

## Discussion

In this paper, we describe a study protocol to evaluate the systems-level implementation of a stepped-care model for identifying and treating help-seeking youth at CHRP across six CMH agencies in Sacramento County, CA. This intervention is novel in the US, where CHRP services are typically offered in specialized early psychosis services, and thus, stepped care that begins at the point of first contact with the mental health system is rare. Further, empirical guidance about which specific interventions for CHRP are effective in community settings is inconclusive. To close this critical gap, this effectiveness-implementation hybrid study aims to implement and evaluate the applicability and effectiveness of a community-based, stepped-care program within a complex healthcare system. There are several notable strengths of the SCIP-Step Program. First, the unique and innovative partnership between CMH agencies and the academic medical center (i.e., UCD Early Psychosis Programs) creates a collaborative relationship between clinical and dissemination teams that allows for community partnership in the development of implementation plans. This approach is critical to demonstrate that an intervention program can work in real-world settings to further reduce the research-practice gap. Second, all CMH agencies involved in the SCIP-Step Program serve those with either public insurance (Medi-Cal) or no insurance. This partnership further expands access to care in underserved communities. Third, locating assessment and stepped care at generalist mental health clinics that are typically first points of help-seeking and are embedded within the communities that they treat moves the initial identification phase of stepped care closer to the community population level. Treating people within their own communities, rather than after they are referred to specialty early psychosis services, simplifies pathways to care and may support provision of services by providers who are more representative of the populations with whom they work. This placement also increases knowledge about CHRP and capacity to effectively treat youths who meet criteria in the general mental health workforce. Finally, a large, diverse sample and longitudinal outcome tracking at multiple time points allows us to generate meaningful findings, both statistically and clinically. Results will inform the applicability and effectiveness of the innovate stepped-care model (e.g., whether recipients of care support acceptability and show clinical improvements, whether clinicians feel it is an appropriate and effective treatment modality that they can implement within their busy clinic, etc.). Important future directions include 1) developing strategies to maintain and sustain these clinical programs without external funding or early psychosis specialist support, 2) more formally evaluating the effectiveness of the stepped-care model and CBCM, and 3) scaling up such stepped care to further reduce barriers to accessing care in the CHRP population.

## Data Availability

No datasets were generated or analysed during the current study.

## Abbreviations

ANOVA: Analysis of Variance
APS: Attenuated psychosis syndrome
ARMS: At-risk mental state
CATS: Child and Adolescent Trauma Screen
CBCM: Cognitive Behavioral Case Management
CBT: Cognitive-Behavioral Therapy
CHRP: Clinical high-risk for psychosis
CMH: Community mental health
CSC: Coordinated specialty care
DSM-5: Diagnostic and Statistical Manual of Mental Disorders, Fifth Edition
DUP: Duration of untreated psychosis
EDAPT: Early Diagnosis and Preventive Treatment Clinic (UC Davis Early Psychosis Program)
EPINET: Early Psychosis Intervention Network
EBP: Evidence-based practices
FIT: Flexible Integrated Treatment
FEP: First episode of psychosis
FFT: Family-Focused Therapy
GFR: Global Functioning Role
GFS: Global Functioning Social
HIPAA: Health insurance portability and accountability act
IQR: Inter-quartile range
IRB: Institutional Review Board
LEC: Life Events Checklist
MCSI: Modified Colorado Symptom Index
Mini-SIPS: Abbreviated Clinical Structured Interview for DSM-5 Attenuated Psychosis Syndrome
NOMS: National Outcome Measures
PCL-5: PTSD Checklist for DSM-5
PQ-B: Prodromal Questionnaire-Brief
PTSD: Posttraumatic stress disorder
QI: Quality improvement
Q&A: Question and Answer
REDCap: Research Electronic Data Capture
RE-AIM: Reach, Effectiveness, Adoption, Implementation, and Maintenance
SAMHSA: Substance Abuse and Mental Health Services Administration
SCIP-Step: Sacramento Clinical high-risk Intervention for Psychosis Stepped Care Program
SCBHS: Sacramento County Department of Health Services - Behavioral Health Services
SCORE-15: Systemic Clinical Outcome and Routine Evaluation - 15 item
SIPS: Structured Interview for Psychosis-risk Syndromes
SD: Standard deviation
STEP: Staged Treatment in Early Psychosis
TF-CBT: Trauma-Focused Cognitive Behavioral Therapy
UCD: University of California, Davis
US: United States
